# *In vitro* drug testing based on contractile activity of C2C12 cells in an epigenetic drug model

**DOI:** 10.1038/srep44570

**Published:** 2017-03-16

**Authors:** Kazushi Ikeda, Akira Ito, Ryusuke Imada, Masanori Sato, Yoshinori Kawabe, Masamichi Kamihira

**Affiliations:** 1 Graduate School of Systems Life Sciences, Kyushu University, 744 Motooka, Nishi-ku, Fukuoka 819-0395, Japan; 2 Department of Chemical Engineering, Faculty of Engineering, Kyushu University, 744 Motooka, Nishi-ku, Fukuoka 819-0395, Japan

## Abstract

Skeletal muscle tissue engineering holds great promise for pharmacological studies. Herein, we demonstrated an *in vitro* drug testing system using tissue-engineered skeletal muscle constructs. In response to epigenetic drugs, myotube differentiation of C2C12 myoblast cells was promoted in two-dimensional cell cultures, but the levels of contractile force generation of tissue-engineered skeletal muscle constructs prepared by three-dimensional cell cultures were not correlated with the levels of myotube differentiation in two-dimensional cell cultures. In contrast, sarcomere formation and contractile activity in two-dimensional cell cultures were highly correlated with contractile force generation of tissue-engineered skeletal muscle constructs. Among the epigenetic drugs tested, trichostatin A significantly improved contractile force generation of tissue-engineered skeletal muscle constructs. Follistatin expression was also enhanced by trichostatin A treatment, suggesting the importance of follistatin in sarcomere formation of muscular tissues. These observations indicate that contractility data are indispensable for *in vitro* drug screening.

Tissue-engineered skeletal muscle constructs have attracted attention for regenerative therapy^1^, basic biological studies^2^, bio-actuators^3^, as a meat alternative^4^, and for drug discovery^5^. *In vitro* culture systems have been applied for drug discovery for patients with injured, diseased and age-related muscle dysfunction^6^,^7^. Conventionally, two-dimensional (2D) cell culture systems have been used to elucidate the efficacy of new drugs by examining their effect on the formation of myotubes^8^. However, 2D cell culture systems are limited in their ability to mimic *in vivo* skeletal muscle function, mainly due to lack of native muscle architecture. One of the most important characteristics of skeletal muscle is its ability for force generation. Thus, artificial skeletal muscle constructs should mimic the architecture of native muscle and exhibit contractile force generation. Using primary myoblasts and/or a C2C12 myoblast cell line, several researchers have developed three-dimensional (3D) culture systems that are available for contractile force measurement^9^,^10^, and tissue-engineered skeletal muscle constructs have been applied in drug testing^11^,^12^, atrophy modeling^7^,^13^ and muscular disease modeling^6^. In a previous study, we developed a method for fabricating functional skeletal muscle tissue constructs using a magnetic force-based tissue engineering (Mag-TE) technique^14^,^15^, in which C2C12 cells were labeled with magnetite cationic liposomes (MCLs)^16^ as a functional magnetic nanoparticle, and assembled by applying a magnetic field to form a cell-dense and aligned fascicle-like structure^15^.

Here, we describe a tissue-engineered skeletal muscle culture system for drug testing based on contractility data presented in force generation. In the present study, tissue-engineered skeletal muscle constructs fabricated by Mag-TE were exposed to small-molecule drugs known to enhance myotube formation. The myogenic effects of small-molecule drugs were compared between 2D cell culture and 3D tissue culture. Small molecules (< 1000 Da) drugs that cause epigenetic changes, such as histone deacetylase (HDAC) inhibitors and DNA methyltransferase (DNMT) inhibitors, have the potential to promote myogenic differentiation. The stimulatory effect of histone acetyltransferases on gene expression is inhibited by HDACs that promote chromatin condensation and thereby repress transcription. HDAC inhibitors promote myogenesis because they inhibit class I and class II HDACs, which repress MyoD and myocyte enhancer factor (MEF) 2 activity, respectively^17^,^18^. Iezzi *et al*. were the first to show that HDAC inhibitors, including trichostatin (TSA), valproic acid (VPA) and sodium butyrate (SB), promoted muscle differentiation in *in vitro* 2D cell culture and *in vivo* mouse embryos^19^. Conversely, the relationship between epigenetic modification and myogenesis was first observed when C3H10T1/2 embryonic fibroblasts were treated with a DNMT inhibitor, 5-azacytidine (5AC)^20^. Upon exposure to 5AC, the promoters of muscle regulatory factors (MRFs), including MyoD, were demethylated during myogenic differentiation^21^. Hupkes *et al*. reported that the treatment of C2C12 cells with 5AC induced genome-wide demethylation and upregulation of muscle-specific genes, resulting in enhanced myogenesis in 2D cell culture^22^. In the present study, four different HDAC inhibitors with three types of chemical structure [the hydroxamic acid TSA, the short-chain fatty acids VPA and SB, and the cyclic tetrapeptide apicidin (API)] and a DNMT inhibitor (5AC) were used for drug testing.

The therapeutic potential of HDAC inhibitors for muscular dystrophy has been previously confirmed, with their therapeutic effect partly relying on a myostatin antagonist follistatin^23^. Follistatin expression is regulated by class I HDACs, which inhibit MyoD activity^24^. Iezzi *et al*. reported that muscles from mice treated with HDAC inhibitors exhibited elevated production of follistatin, indicating follistatin as a central mediator of the myogenic effects exerted by HDAC inhibitors on skeletal muscles^23^. Therefore, we further investigated the effects of follistatin overexpression in C2C12 cells on myogenic differentiation, sarcomere formation and force generation of artificial skeletal muscle constructs.

## Results

### Effects of epigenetic drugs on myogenic differentiation in 2D culture

**Figure 1:**
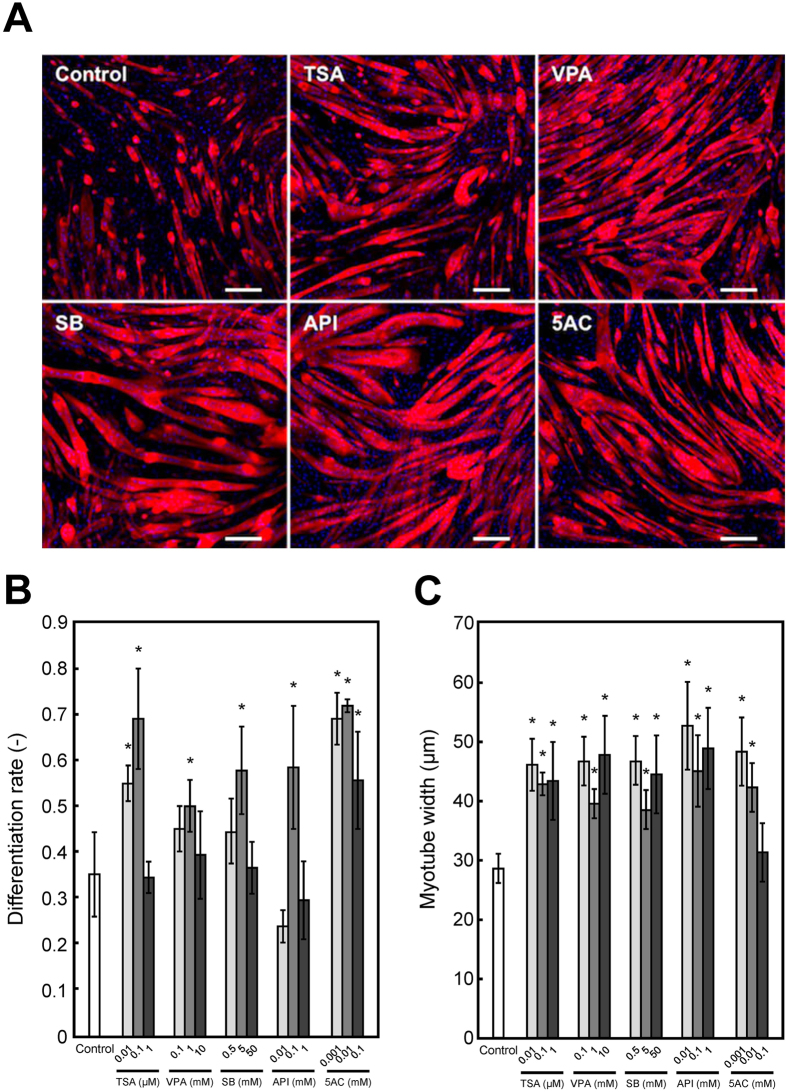
Effects of epigenetic drugs on the myogenic differentiation of C2C12 cells in two-dimensional culture. (**A**) Fluorescence microscopy images of myotubes at day 7. C2C12 cells were treated with trichostatin A (TSA, 0.1 μM), valproic acid (VPA, 1 mM), sodium butyrate (SB, 5 mM), apicidin (API, 0.1 mM) or 5-azacytidine (5AC, 0.01 mM) at day 4. Red, α-actinin-positive myotubes; blue, DAPI-positive nuclei. Scale bar, 200 μm. (**B**) Quantitative image analysis of myotube differentiation rate. The data are expressed as mean ± SD. *P < 0.05 vs. control. (**C**) Quantitative image analysis of myotube width. The data are expressed as mean ± SD of triplicate experiments. *P < 0.05 vs. control.

To investigate the effects of epigenetic drugs on myogenic differentiation and hypertrophy of 2D cultured myoblasts, differentiating C2C12 cells were exposed to TSA, VPA, SB, API or 5AC. Immunofluorescent staining with anti-α-actinin antibody revealed that C2C12 myotubes were formed by fusion of single nucleated cells in response to differentiation medium (Fig. 1A). C2C12 cells treated with TSA (0.01 and 0.1 μM), VPA (1 mM), SB (5 mM), API (0.1 mM) and 5AC (0.001, 0.01 and 0.1 mM) significantly (*P *< 0.05) increased the differentiation rate compared with the control (Fig. 1B). Similarly, myogenic hypertrophy, which is the augmentation of skeletal muscle through an increase in size of myotubes, was induced in C2C12 myotubes by TSA (0.01, 0.1 and 1 μM), VPA (0.1, 1 and 10 mM), SB (0.5, 5 and 50 mM), API (0.01, 0.1 and 1 mM) and 5AC (0.001 and 0.01 mM) (Fig. 1C).

### Effects of epigenetic drugs on contractile force generation of tissue-engineered skeletal muscle constructs

**Figure 2:**
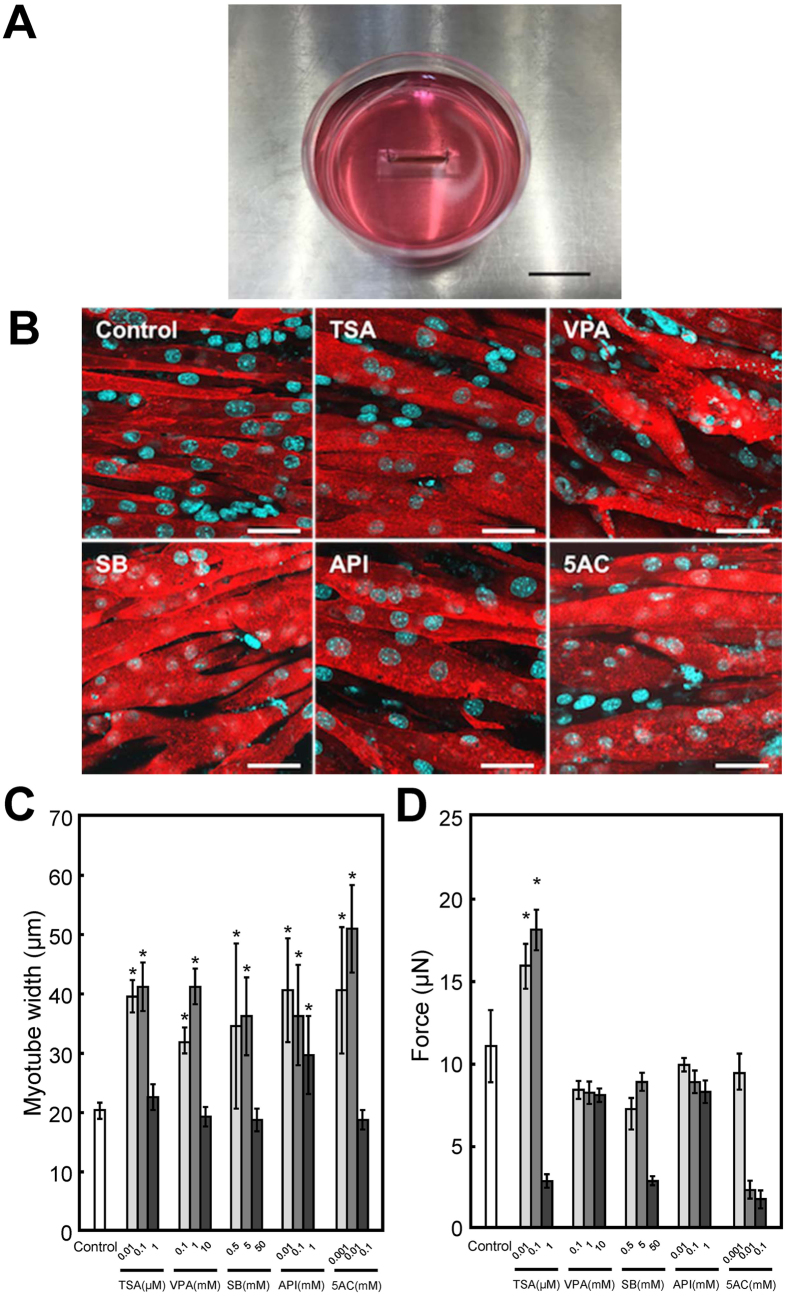
Effects of epigenetic drugs on force generation of tissue-engineered skeletal muscle constructs. (**A**) Macroscopic image of the skeletal muscle constructs fabricated using the Mag-TE technique. Scale bar, 10 mm. (**B**) Fluorescence microscopy images of α-actinin-positive myotubes (red) in the tissue-engineered skeletal muscle tissue constructs at day 7. Nuclei were stained using DAPI (blue). Tissue-engineered skeletal muscle tissue constructs were treated with trichostatin A (TSA; 0.1 μM), valproic acid (VPA; 1 mM), sodium butyrate (SB; 5 mM), API (0.1 mM) or 5-azacytidine (5AC; 0.01 mM) at day 4. Scale bars, 50 μm. (**C**) Quantitative image analysis of myotube width in tissue-engineered skeletal muscle tissue constructs. The data are expressed as mean ± SD of three constructs. *P < 0.05 vs. control. (**D**) The force generated by tissue-engineered skeletal muscle tissue constructs on day 7. The data are expressed as mean ± SD of three constructs. *P < 0.05 vs. control.

**Figure 3:**
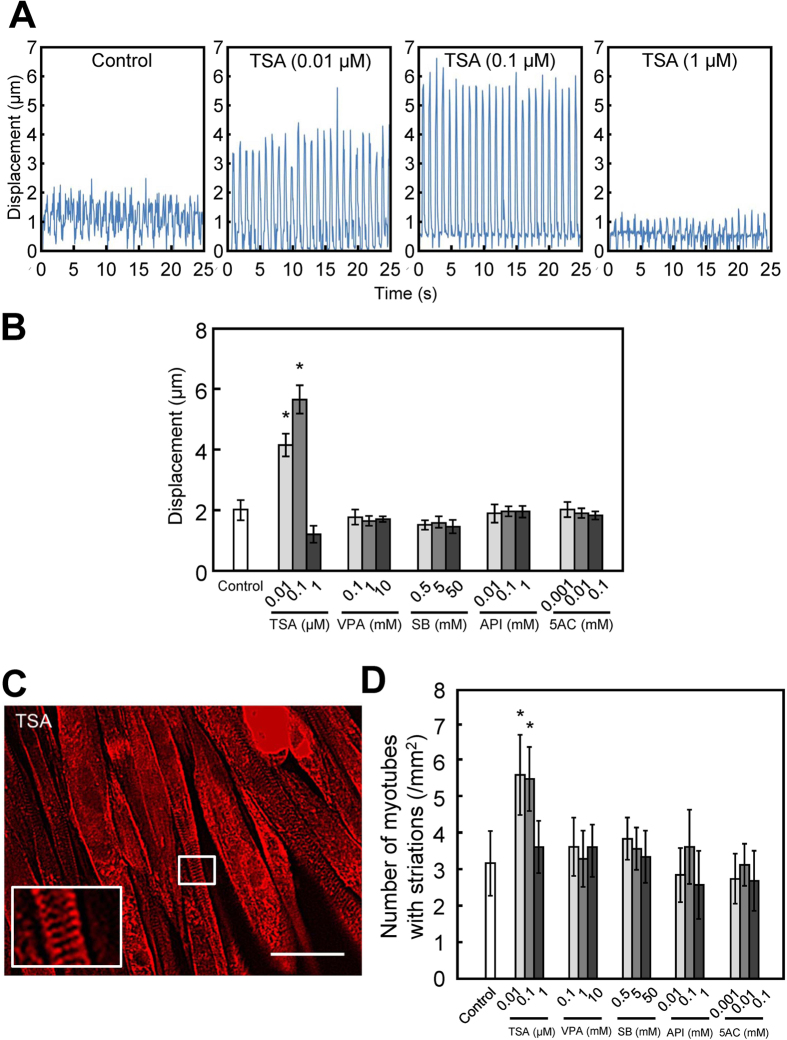
Effects of epigenetic drugs on the contractile activity of C2C12 myotubes in two-dimensional culture. (**A**) Contractile movement of myotubes. C2C12 cells were treated with trichostatin A (TSA; 0.01, 0.1 or 1 μg/ml) on day 4. Electrical pulse stimulation (voltage, 0.3 V/mm; width, 10 ms; frequency, 1 Hz) was applied, and the displacement of a single cell within the myotubes was analyzed. (**B**) Quantitative analysis of the range of displacement. The data are expressed as mean ± SD of triplicate experiments. *P < 0.05 vs. control. (**C**) A representative fluorescence microscopy image of a myotubes (treated 0.1 μM of TSA) with striations of sarcomeric α-actinin (red). Scale bar, 50 μm. Inset: Magnified image of area in white rectangle. (**D**) Quantitative image analysis of the number of myotubes with striations. The number of myotubes displaying striations of sarcomeric α-actinin was quantified by counting myotubes. The data are the means ± SD of triplicate experiments. *P < 0.05 vs. control.

We fabricated 3D tissue constructs of C2C12 cells using the Mag-TE technique (Fig. 2A), to investigate the effects of epigenetic drugs on contractile force generation. As shown in Fig. 2B, the outer cell layer of the tissue constructs was composed of dense and oriented myotubes. Since myogenic hypertrophy is generally involved in contractile force generation of skeletal muscle *in vivo*, myotube width was measured in the tissue constructs. Consistent with the results of the 2D cell culture (Fig. 1C), myotube width of C2C12 cells in 3D tissue constructs was significantly increased by TSA (0.01 and 0.1 μM), VPA (0.1 and 1 mM), SB (0.5 and 5 mM), API (0.01, 0.1 and 1 mM) and 5AC (0.001 and 0.01 mM) (Fig. 2C). The maximum twitch forces produced by tissue-engineered skeletal muscle constructs are shown in Fig. 2D. Interestingly, contractile force generation was significantly (*P* < 0.05) enhanced by TSA (0.01 and 0.1 μM) treatment (Fig. 2D); however, myotube width, in both 2D cell culture (Fig. 1C) and 3D tissue culture (Fig. 2C), was increased by all epigenetic drugs tested.

### Two-dimensional contractile activity of myotubes exposed to epigenetic drugs

To clarify the effects of epigenetic drugs on myogenic differentiation and contractile force generation, 2D contractile activity was investigated by examining myotube movement in response to electrical pulse stimulation (EPS). On day 7 of culture, myotubes displayed contractile responses to EPS of 0.3 V/mm for 4 ms at 1 Hz. Figure 3A shows the contractile profile of C2C12 myotubes treated with TSA. Control myotubes displayed a small range of displacement and arrhythmic contraction, while myotubes treated with TSA (0.01 and 0.1 μM) contracted rhythmically in response to EPS, with a dynamic range of displacement. Consistent with the results of contractile force generation in 3D tissue culture (Fig. 2D), the contractile activity of myotubes in 2D cell culture was enhanced by TSA (0.01 and 0.1 μM) treatment (Fig. 3B), suggesting that 2D contractile activity partially mimics the contractile force generation capability of 3D tissue constructs. EPS for 20 min induced the formation of striated patterns on myotubes (Fig. 3C). TSA (0.01 and 0.1 μM) treatment resulted in an obvious striated pattern consisting of sarcomeric α-actinin (Fig. 3C) and an increase in the number of myotubes with striations (Fig. 3D). Consistent with the results of myotube displacement (Fig. 3B), TSA (0.01 and 0.1 μM) treatment increased the number of myotubes with striations (Fig. 3D). These results indicate that there is a high correlation between sarcomere formation as well as contractile activity in 2D cell culture and contractile force generation of 3D skeletal muscle tissue constructs.

### Effects of follistatin expression on TSA-treated myotubes in 2D cell culture

**Figure 4:**
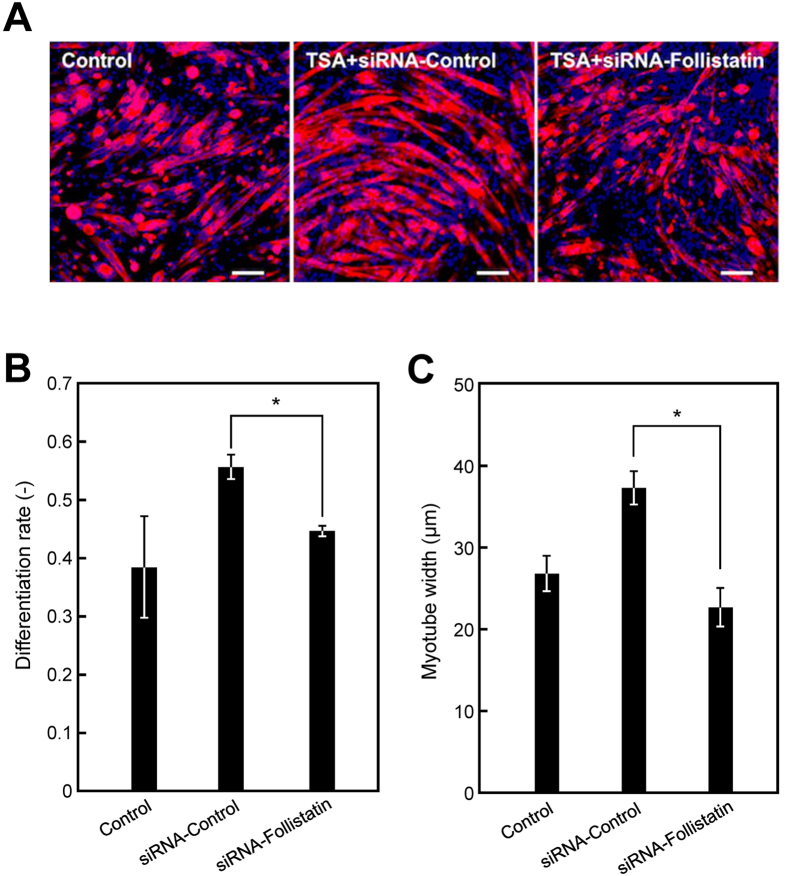
Role of follistatin expression in myotube differentiation in two-dimensional culture. (**A**) Fluorescence microscopy images of α-actinin-positive myotubes (red) at day 7. Nuclei were stained using DAPI (blue). C2C12 cells were treated with trichostatin A (TSA; 0.1 μM) at day 4. siRNAs for follistatin (siRNA-follistatin) or a negative control (siRNA-Control) were transfected at day 5. Scale bars, 200 μm. (**B**) Quantitative image analysis of myotube differentiation rate. The data are expressed as mean ± SD of triplicate experiments. *P < 0.05. (**C**) Quantitative image analysis of myotube width. The data are expressed as mean ± SD of triplicate experiments. *P < 0.05.

TSA enhanced force generation of artificial skeletal muscle tissues (Fig. 2D). To elucidate the mechanism, we investigated whether follistatin expression was responsible for the enhanced muscular functions of skeletal tissues treated with TSA. First, endogenous follistatin in C2C12 myotubes was suppressed by siRNA. Treatment of siRNA-follistatin counteracted the effects of TSA on myogenic differentiation (Fig. 4A), including the increased differentiation rate (Fig. 4B) and myotube width (Fig. 4C), suggesting that follistatin is a key mediator in the myogenic events exerted by TSA treatment.

### Effects of follistatin gene transfer on muscular functions

**Figure 5:**
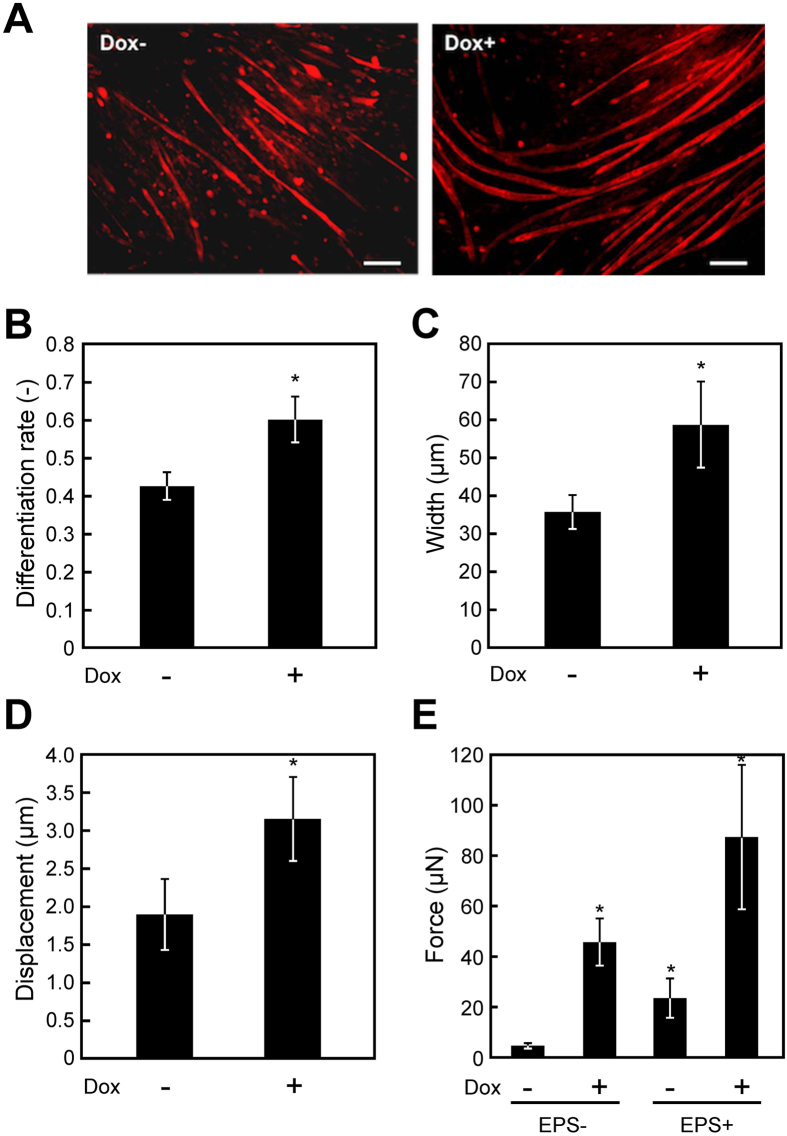
Effects of follistatin gene transfer on muscular functions. (**A**) Fluorescence microscopy images of α-actinin-positive myotubes (red) of C2C12/FST cells at day 7 in two-dimensional (2D) culture. Nuclei were stained using DAPI (blue). C2C12/FST cells were cultured in the presence (Dox + ) or absence (Dox−) of doxycycline (Dox) for 7 days. Scale bars, 200 μm. (**B**) Quantitative image analysis of myotube differentiation rate at day 7 in 2D culture. The data are expressed as mean ± SD of triplicate experiments. *P < 0.05 vs. Dox−. (**C**) Quantitative image analysis of myotube width at day 7 in 2D culture. The data are expressed as mean ± SD of triplicate experiments. *P < 0.05 vs. Dox−. (**D**) Contractile movement of myotubes in 2D culture. Electrical pulse stimulation (EPS; voltage, 0.3 V/mm; width, 10 ms) was applied at 1 Hz, and the displacement of a single point was analyzed within the myotubes on day 7. Quantitative analysis of displacement was performed. The data are expressed as mean ± SD of triplicate experiments. *P < 0.05 vs. Dox−. (**E**) The force generated by tissue-engineered skeletal muscle tissue constructs on day 7. Tissue-engineered skeletal muscle constructs were cultured with (EPS + ) or without (EPS−) continuous EPS at an amplitude of 0.3 V/mm, width of 4 ms, and frequency of 1 Hz during tissue culture. The data are expressed as mean ± SD of three constructs. *P < 0.05 vs. EPS−Dox−.

To verify the involvement of follistatin in myogenic differentiation and contractile force generation, we created genetically-engineered C2C12 cells capable of Dox-inducible follistatin expression (C2C12/FST). The expression levels of C2C12/FST cells in the absence and presence of Dox were 3.9 and 6.6 fg/cell, respectively. In 2D cell culture, immunofluorescent staining with anti-α-actinin antibody (Fig. 5A) revealed that C2C12/FST myotubes exhibited an enhanced differentiation rate (Fig. 5B) and increased myotube width (Fig. 5C) in the presence of Dox. Moreover, the contractile activity of C2C12/FST myotubes indicated a significant increase of displacement in the presence of Dox (Fig. 5D). Finally, artificial skeletal muscle tissue constructs comprised of C2C12/FST cells were fabricated using Mag-TE, and contractile force generation of the tissue constructs was measured. With Dox treatment, the contractile force generated by C2C12/FST tissue constructs was 9.1 times higher than that without Dox treatment (Fig. 5E). EPS culture, in which C2C12/FST cells were cultured for 7 days in differentiation medium with continuous EPS at amplitude of 0.3 V/mm, width of 4 ms, and frequency of 1 Hz, further enhanced the contractile force generated by C2C12/FST tissue constructs. In addition, Dox-induced C2C12/FST tissue constructs cultured with EPS had 17.1 times higher contractile force generation than C2C12/FST tissue constructs cultured without Dox or EPS (Fig. 5E). These results indicate that follistatin has a crucial role in the expression of muscular functions, including myogenic differentiation and contractile force generation.

## Discussion

In this study, the utility of tissue-engineered skeletal muscle constructs for drug testing was evaluated by measuring contractile force generation in response to exposure to epigenetic drugs. The use of established myoblast cell lines, including mouse C2C12 cells, has numerous benefits for drug screening and testing, as they provide repeatable and reproducible systems, which reduces the number of animal studies. Additionally, drug screening using tissue-engineered skeletal muscle constructs should serve as a useful secondary screening method that could reduce the time, cost and the number of animals required. In this study, we fabricated tissue-engineered skeletal muscle constructs using the Mag-TE method as follows: (1) MCL-labeled C2C12 cells were seeded into a well with a polycarbonate cylinder fixed to the center; (2) cells were uniformly accumulated at the bottom of the well by applying a magnetic force; (3) a sheet-like structure contracted to form a ring-like tissue construct surrounding the cylinder; (4) the tissue ring was coated with extracellular matrix and detached from the cylinder; (5) the tissue ring was cultured in the differentiation medium to form fascicle-like tissues. During the differentiation culture, some tissues were ruptured because of their high degree of compaction; however, tissues remained more than 95% intact at day 7. Tissue fabrication was performed in a 24-well tissue culture plate, allowing for 24 tissue-engineered skeletal muscle constructs to be successfully prepared in one procedure. The tissue-engineered skeletal muscle constructs showed high reproducibility in contractile force generation. Taken together, this system of using tissue-engineered skeletal muscle constructs fabricated by Mag-TE has proven to be robust and highly reproducible.

Consistent with previous reports, the exposure of C2C12 cells to HDAC inhibitors (TSA, VPA or SB)^19^ or a DNMT inhibitor (5AC)^22^ induced a dramatic increase in the formation of myotubes in 2D culture (Fig. 1). We found that API also enhanced myogenic differentiation. As a HDAC inhibitor, it was reported that API inhibited the proliferation of tumor cells via the induction of p21^25^. During myogenic development, an appropriate balance between proliferation and differentiation of muscle progenitor cells is essential for the maturation of skeletal muscle^26^. Because MyoD induces cell cycle arrest during myogenic differentiation via inducing p21 expression^27^, API may also enhance myogenic differentiation via p21 upregulation.

Drug screening based on contractile force generation of tissue-engineered skeletal muscle constructs is a promising approach; however, there have been very few reports using a skeletal muscle tissue culture system for drug testing based on measuring contractility. Vandenburgh *et al*. reported that 0.2 μM TSA increased contractile force generation of skeletal muscle tissue constructs derived from *mdx* murine myoblasts, and the results showed a relatively narrow range of effective concentration and cytotoxicity at higher concentrations^6^, which is consistent with our results (Fig. 2D). In contrast, other epigenetic drugs tested in this study exhibited no positive effect on contractile force generation at all concentrations used (Fig. 2D); however, myogenic differentiation was enhanced in both 2D cell culture (Fig. 1B,C) and 3D tissue culture (Fig. 2C). We believe that this is the first report comparing the effects of drugs on myogenic differentiation in 2D culture and the contractile force generation of 3D tissue-engineered skeletal muscle. Our results suggest that morphological analysis using myogenic differentiation markers alone is not sufficient for drug testing. Contractile force generation capability reflects not only the extent of differentiation, including the expression of MRFs, but also the niche, including the ECM, and the architecture, including sarcomere formation. In the present study, TSA increased the number of myotubes displaying striation of sarcomeric α-actinin (Fig. 3D), along with enhanced 2D contractile activity (Fig. 3B), and these parameters were highly correlated with contractile force generation of tissue-engineered skeletal muscle constructs (Fig. 2D). As a conclusion, we believe that both 2D (contractile activity) and 3D (force generation) constructs can be used for drug testing. The evaluation of sarcomere formation and/or contractile activity in 2D cultured myotubes is particularly advantageous for high-throughput and high-content drug screening. In contrast, drug testing based on force generation of artificial skeletal muscle tissues, mimicking the architecture of native muscles, is more effective for quantitative evaluation of drugs, because unidirectionally aligned myotubes in the tissue construct efficiently exert a contractile force that enables quantitative measurement of force generation.

Mal *et al*. reported that follistatin expression is upregulated by HDAC inhibitors^24^. To elucidate the mechanism of enhanced contractile force generation in TSA-treated tissue constructs, we examined follistatin expression after TSA treatment. TSA is one of the most potent HDAC inhibitors in myoblasts, and several groups have reported the effects of TSA on myogenic differentiation^23^,^28^. Using microarray analysis, Iezzi *et al*. reported that follistatin is one of the most highly upregulated transcripts after TSA treatment of C2C12 cells^23^. Follistatin, a glycoprotein that interacts with the transforming growth factor (TGF)-β superfamily, is essential for skeletal muscle development and growth^29^. Follistatin was originally reported as an antagonist of myostatin; myostatin induces myotube atrophy via inactivation of insulin-like growth factor (IGF)/phosphoinositide 3-kinase (PI3K)/Akt signaling^30^, and enhances the degradation of sarcomeric proteins^31^. Additionally, follistatin was shown to increase muscle mass and strength through the Smad3/Akt/mammalian target of rapamycin (mTOR) signaling pathway, independent of myostatin^32^. Iezzi *et al*. reported that VPA and SB increased follistatin expression^23^. In the present study, however, no significant increase in follistatin was observed in VPA- or SB-treated C2C12 myotubes (Supplementary Figure 1), suggesting that TSA is the most potent HDAC inhibitor among them. Conversely, a significant increase in follistatin expression was also observed in C2C12 myotubes treated with API and 5AC (Supplementary Figure 1). Fan *et al*. reported that 5AC (0.01 mM) increased follistatin expression in porcine satellite cells^33^, which is consistent with our results. These results suggest that these small-molecule drugs induce epigenetic changes in various genes during myogenic differentiation and that follistatin is not the sole effector involved in contractile force generation. Although the detailed mechanism remains unclear, follistatin signaling pathways are partly responsible for increased contractile force generation of TSA-treated muscles. Furthermore, we evaluated the effects of follistatin gene transfer in C2C12 cells on myogenic differentiation. C2C12/FST cells exhibited increased differentiation rate (Fig. 5B), myotube width (Fig. 5C), and contractile activity (Fig. 5D) in 2D cell culture. The contractile force generation of tissue constructs was also dramatically enhanced by follistatin gene transfer (Fig. 5E). Collectively, these data indicate that follistatin is a key mediator of contractile force generation. In our previous study, we demonstrated that EPS culture, functioning as “physical training”, enhanced force generation of artificial skeletal muscle tissues^34^. These results prompted us to apply EPS culture for C2C12/FST cells, in which we successfully achieved a 17-fold increase in contractile force generation compared with control tissue constructs (Fig. 5E). In the previous study, we showed that EPS culture induced sarcomere formation in tissue-engineered skeletal muscle constructs^34^. Interestingly, in the present study, overexpression of follistatin gene induced sarcomere formation in tissue-engineered skeletal muscle constructs, even without EPS culture (Supplementary Figure 2). These results suggest that follistatin is a promising therapeutic target for improving muscle functions.

In conclusion, we demonstrated the feasibility of an *in vitro* system for drug testing using tissue-engineered skeletal muscle constructs fabricated by the Mag-TE technique. Our observations may provide important insight for *in vitro* drug screening. Most importantly, we demonstrated that contractility data is indispensable for *in vitro* drug discovery and pharmacological studies.

## Methods

### Epigenetic drug treatment

Mouse myoblast C2C12 cells^35^ were cultured as described previously^36^. C2C12 cells were exposed to TSA (0.01, 0.1 or 1 μM; Wako Pure Chemical Industries, Osaka, Japan), VPA (0.1, 1 or 10 μM; Wako Pure Chemical Industries), SB (0.5, 5 or 50 μM; Wako Pure Chemical Industries), API (0.01, 0.1 or 1 μM; Enzo Life Sciences, NY, USA) or 5AC (0.001, 0.01 or 0.1 mM; Wako Pure Chemical Industries) for 24 h, 4 days after the induction of differentiation (day 4). The following day, the medium was replaced with fresh medium to remove the small-molecule drugs.

### Measurement of differentiation rate and myotube width

Immunofluorescent staining was performed according to a previous study^36^. Myogenic differentiation rate and myotube width were determined at day 7. Micrographs of five fields, in each of three separate wells per sample (2D cell culture) or in each of three tissue constructs (3D tissue culture), were randomly captured. The α-actinin-positive myotube width and the number of 4′,6-diamidino-2-phenylindole (DAPI)-stained nuclei were measured using a BZ-9000 microscope (Keyence). The differentiation rate was defined as follows: Differentiation rate = (the number of DAPI-stained nuclei in α-actinin-positive myotubes in a field) / (the number of DAPI-stained nuclei in a field). For estimating the mean value of myotube width, the five largest myotubes in five fields for each of three separate wells per sample (2D cell culture) or for each of three tissue constructs (3D tissue culture) were measured.

### Fabrication of artificial skeletal muscle tissues using a Mag-TE technique

Artificial skeletal muscle tissues were fabricated with the magnetic force-based tissue engineering (Mag-TE) method^36^. Briefly, magnetite cationic liposomes (MCLs) were prepared from colloidal magnetite (Fe_3_O_4_; average particle size, 10 nm) and a lipid mixture consisting of *N*-(α-trimethylammonioacetyl)-didodecyl-D-glutamate chloride, dilauroylphosphatidylcholine, and dioleoylphosphatidylethanolamine, in a molar ratio of 1:2:2. To label C2C12 cells magnetically, 3 × 10^6^ cells were seeded in 100-mm tissue culture dishes containing 10 mL growth medium in the presence of MCLs (net magnetite concentration, 100 pg/cell) and incubated for 6 h to allow MCL uptake. A collagen solution was prepared by mixing type I collagen solution (Nitta Gelatin, Osaka, Japan), DMEM and neutralization buffer (0.05 M NaOH) at a volume ratio of 8:1:1. An MCL-labeled cell suspension (1 × 10^6^ cells in 50 μL) was mixed with an ECM precursor solution composed of collagen solution (70 μL; final concentration, 0.5 mg/mL), FBS (15 μL) and Matrigel basement matrix (15 μL; BD Biosciences). Subsequently, the mixture was added into wells of a 24-well ultra-low attachment plate (150 μL/well, Corning, New York, NY, USA) with a polycarbonate cylinder (diameter, 12 mm; height, 5 mm) fixed at the center of each well, and a neodymium magnet (diameter, 30 mm; magnetic induction, 0.4 T) was placed under the well. Thereafter, growth medium was added to each well. One day after cell seeding, the cell layer shrunk around the cylinder, resulting in the formation of a ring-shaped artificial skeletal muscle tissue construct. The cellular ring was removed from the cylinder and hooked around two stainless-steel pins (Shiga, Tokyo, Japan) that were positioned 10 mm apart from one another. The cellular tissues were cultured using differentiation medium (day 0) in wells of a 6-well culture plate for 7 days, to induce myogenic differentiation to fabricate skeletal muscle tissue constructs (The average diameter, 500 μm).

### Measurement of contractile force generation

Two carbon electrodes were arranged 18 mm apart at opposite sides of a well of a 4-well tissue culture plate. A tissue-engineered skeletal muscle tissue construct was hooked around two stainless-steel pins. One pin was attached to a force transducer (AE-801; SensorOne, Sausalito, CA, USA) and the other was fixed to a silicone sheet placed on the bottom of a well of the culture plate. Electrical pulse generation was controlled with specially-designed LabView software (National Instruments, Austin, TX, USA). For measuring twitch contractions, the tissue samples were stimulated with an electrical pulse of 18 V and a width of 10 ms.

### Myotube contractile activity assay

C2C12 cells (5 × 10^4^) were seeded in 35-mm tissue culture dishes on day −3. The medium was replaced with differentiation medium on day 0. On day 7, an electrical pulse stimulation was applied to the cells. Carbon electrodes were placed 18 mm apart at opposite sides of a tissue culture dish. The generation of electric pulses was controlled by a function generator (NF Corporation, Kanagawa, Japan). The cells were stimulated with an electric pulse for 30 min with the following properties: voltage, 0.3 V/mm; width, 4 ms; frequency, 1 Hz. After 30 min of EPS, the electric pulses were applied again and myotube movement was recorded at a rate of 15 frames/s for 25 s at three positions on the bottom surface in each of three separate dishes using a BZ-9000 microscope (Keyence). To estimate the range of displacement, three myotubes displaying the highest contractile activity in each of three fields in three separate dishes were measured using motion analyzer software (Keyence).

To visualize striation patterns in myotubes, C2C12 cells (5 × 10^4^) were seeded into 35-mm glass bottom dishes (Code 3970-035, Asahi Techno Glass) and α-actinin staining was performed at day 7. Microscopic images of five fields in each of three separate wells per sample were captured under a BZ-9000 fluorescence microscope (Keyence) and processed by using BZ-Analyzer software (Keyence), and the number of myotubes possessing sarcomeric α-actinin striations was counted.

### RNA interference

C2C12 cells were transfected with an oligonucleotide for follistatin siRNA (Silencer siRNA, 50 μM; Cat# s66250, Thermo Fisher Scientific, Waltham, MA, USA) or Silencer Negative Control #1 siRNA (50 μM; Cat# AM4611, Thermo Fisher Scientific) at day 5 using Endo-Porter (Gene Tools, Philomath, OR, USA), according to the manufacturer’s protocols. The Silencer siRNA is designed for maximum potency and specificity using a highly effective and extensively tested algorithm.

### Follistatin gene expression system

293FT cells were used as a producer of retroviral vectors based on the Moloney murine leukemia virus (MoMLV)^37^. MoMLV-derived mouse stem cell virus (MSCV)-based retroviral vectors were used for gene transfer into C2C12 cells. The Tet-On system (Clontech, Mountain View, CA, USA) was incorporated into the retroviral vectors for inducible expression of the follistatin gene. For the construction of pQMSCV/EGFP-TRE-follistatin-WPRE, a follistatin cDNA fragment from pCMV-SPORT6 (Clone ID LIFESEQ95153976; Open Biosystems, Huntsville, AL, USA) was ligated into *Bam*HI-digested pQMSCV/EGFP-TRE-WPRE^37^ to generate pQMSCV/EGFP-TRE-follistatin-WPRE.

Retroviral vectors pseudotyped with the vesicular stomatitis G protein (VSV-G) were produced by transient transfection of 293FT cells with three plasmid DNAs, comprising of a retroviral vector plasmid (pQMSCV/EGFP-CMV-rtTA-WPRE^37^ and pQMSCV/EGFP-TRE-follistatin-WPRE), pcDNA4-gag/pol and pLP/VSV-G^38^, using the lipofection reagent Lipofectamine 2000 (Thermo Fisher Scientific). The culture medium containing viral vector particles was collected, filtered through a 0.45-μm cellulose acetate filter (Advantec, Tokyo, Japan) and used for infection. For the retroviral infection, C2C12 cells (2.5 × 10^5^) were seeded into a 100-mm tissue culture dish (Thermo Fisher Scientific) and cultured for 24 h. Subsequently, the medium was replaced with 10 mL of retroviral solution, containing retroviral vectors encoding rtTA (MSCV/EGFP-CMV-rtTA-WPRE^37^) and follistatin (MSCV/EGFP-TRE-follistatin-WPRE) expression cassettes. The cells were then cultured in the presence of polybrene (8 μg/mL) for 6 h, resulting in the generation of C2C12 cells capable of doxycycline (Dox)-inducible follistatin expression. For all retroviral infections, the viral titers against C2C12 cells were determined by flow cytometry using a FACSCalibur (BD Biosciences, Franklin Lakes, NJ, USA). The viral titers for infection were approximately 2.5 × 10^5^ IU/mL for each vector. Follistatin gene expression was induced by Dox addition (1 μg/ml; Sigma-Aldrich) at day 0, and C2C12 cells or tissues were cultured for 7 days, with daily replacement of the differentiation medium containing Dox (1 μg/ml; Sigma-Aldrich). To measure follistatin expression levels quantitatively, cells were analyzed using a follistatin ELISA kit (cat# DFN00, R&D Systems, Minneapolis, MN, USA) according to the manufacturer’s protocol.

### Electric pulse stimulation during tissue culture

Four days after differentiation induction, the tissue-engineered skeletal muscle constructs in a 6-well plate were placed in a chamber (C-Dish, IonOptix, Milton, MA, USA) to apply EPS. The tissue constructs were placed between two carbon electrodes, 20.3 mm apart from each other. Bidirectional electric pulses were generated using a function generator (NF Corporation), alternator (Matsusada Precision, Shiga, Japan) and amplifier (Apex Microtechnology, Tucson, AZ, USA). Tissue-engineered skeletal muscle constructs were stimulated with continuous electrical pulses at an amplitude of 0.3 V/mm, width of 4 ms, and a frequency of 1 Hz. The differentiation medium was replaced daily.

### Statistical analysis

Statistical comparisons were evaluated using the Mann-Whitney rank sum test, and the values of *P* < 0.05 were considered significantly different.

## Additional Information

**How to cite this article:** Ikeda, K. *et al. In vitro* drug testing based on contractile activity of C2C12 cells in an epigenetic drug model. *Sci. Rep.* **7**, 44570; doi: 10.1038/srep44570 (2017).

**Publisher's note:** Springer Nature remains neutral with regard to jurisdictional claims in published maps and institutional affiliations.

## Supplementary Material

Supplementary Information

## References

[b1] Badylak S. F., Weiss D. J., Caplan A. & Macchiarini P. Engineered whole organs and complex tissues. Lancet. 379, 943–952 (2012). 22405797 10.1016/S0140-6736(12)60073-7

[b2] Bursac N., Juhas M. & Rando T. A. Synergizing engineering and biology to treat and model skeletal muscle injury and disease. Annu. Rev. Biomed. Eng. 17, 217–242 (2015). 26643021 10.1146/annurev-bioeng-071114-040640 PMC4858326

[b3] Feinberg A. W. Biological soft robotics. Annu. Rev. Biomed. Eng. 17, 243–265 (2015). 26643022 10.1146/annurev-bioeng-071114-040632

[b4] Pandurangan M. & Kim D. H. A novel approach for *in vitro* meat production. Appl. Microbiol. Biotechnol. 99, 5391–5395 (2015). 25971200 10.1007/s00253-015-6671-5

[b5] Gibbons M. C., Foley M. A. & Cardinal K. O. Thinking inside the box: keeping tissue-engineered constructs *in vitro* for use as preclinical models. Tissue. Eng. Part B Rev. 19, 14–30 (2013). 22800715 10.1089/ten.TEB.2012.0305

[b6] Vandenburgh H. . Automated drug screening with contractile muscle tissue engineered from dystrophic myoblasts. FASEB J. 23, 3325–3334 (2009). 19487307 10.1096/fj.09-134411 PMC3236595

[b7] Sharples A. P. . Modelling *in vivo* skeletal muscle ageing *in vitro* using three-dimensional bioengineered constructs. Aging Cell. 11, 986–995 (2012). 22882433 10.1111/j.1474-9726.2012.00869.x

[b8] Cross-doersen D. & Isfort R. J. A novel cell-based system for evaluating skeletal muscle cell hypertrophy-inducing agents. In Vitro Cell Dev. Biol. 39, 407–412 (2003). 10.1290/1543-706X(2003)039<0407:ANCSFE>2.0.CO;2 14741040

[b9] Dennis R. G., Kosnik P. E., Gilbert M. E. & Faulkner J. A. Excitability and contractility of skeletal muscle engineered from primary cultures and cell lines. *Am. J*. Physiol. Cell Physiol. 280, C288–C295 (2001). 10.1152/ajpcell.2001.280.2.C288 11208523

[b10] Fujita H., Shimizu K. & Nagamori E. Novel method for fabrication of skeletal muscle construct from the C2C12 myoblast cell line using serum-free medium AIM-V. Biotechnol. Bioeng. 103, 1034–1041 (2009). 19350625 10.1002/bit.22318

[b11] Vandenburgh H. . Drug-screening platform based on the contractility of tissue-engineered muscle. Muscle and Nerve. 37, 438–447 (2008). 18236465 10.1002/mus.20931

[b12] Madden L., Juhas M., Kraus W. E., Truskey G. A. & Bursac N. Bioengineered human myobundles mimic clinical responses of skeletal muscle to drugs. Elife. 4, e04885; 10.7554/eLife.04885 (2015). 25575180 10.7554/eLife.04885 PMC4337710

[b13] Lee P. H. & Vandenburgh H. H. Skeletal muscle atrophy in bioengineered skeletal muscle: a new model system. Tissue. Eng. Part A. 19, 2147–2155 (2013). 23574457 10.1089/ten.TEA.2012.0597

[b14] Yamamoto Y. . Preparation of artificial skeletal muscle tissues by a magnetic force-based tissue engineering technique. J. Biosci. Bioeng. 108, 538–543 (2009). 19914590 10.1016/j.jbiosc.2009.05.019

[b15] Yamamoto Y. . Functional evaluation of artificial skeletal muscle tissue constructs fabricated by a magnetic force-based tissue engineering technique. Tissue. Eng. Part A. 17, 107–114 (2011). 20672996 10.1089/ten.TEA.2010.0312

[b16] Ito A., Shinkai M., Honda H. & Kobayashi T. Medical application of functionalized magnetic nanoparticles. J. Biosci. Bioeng. 100, 1–11 (2005). 16233845 10.1263/jbb.100.1

[b17] McKinsey T. A., Zhang C. L. & Olson E. N. Signaling chromatin to make muscle. Curr. Opin. Cell Biol. 14, 763–772 (2002). 12473352 10.1016/s0955-0674(02)00389-7

[b18] Forcales S. V. & Puri P. L. Signaling to the chromatin during skeletal myogenesis: Novel targets for pharmacological modulation of gene expression. Semin. Cell Dev. Biol. 16, 596–611 (2005). 16129633 10.1016/j.semcdb.2005.07.005

[b19] Iezzi S., Cossu G., Nervi C., Sartorelli V. & Puri P. L. Stage-specific modulation of skeletal myogenesis by inhibitors of nuclear deacetylases. Proc. Natl. Acad. Sci. USA. 99, 7757–7762 (2002). 12032356 10.1073/pnas.112218599 PMC124343

[b20] Taylor S. M. & Jones P. A. Multiple new phenotypes induced in 10T1/2 and 3T3 cells treated with 5-azacytidine. Cell. 17, 771–779 (1979). 90553 10.1016/0092-8674(79)90317-9

[b21] Brunk B. P., Goldhamer D. J. & Emerson C. P. Regulated demethylation of the myoD distal enhancer during skeletal myogenesis. Dev. Biol. 177, 490–503 (1996). 8806826 10.1006/dbio.1996.0180

[b22] Hupkes M. . Epigenetics: DNA demethylation promotes skeletal myotube maturation. FASEB J. 25, 3861–3872 (2011). 21795504 10.1096/fj.11-186122

[b23] Iezzi S. . Deacetylase inhibitors increase muscle cell size by promoting myoblast recruitment and fusion through induction of follistatin. Dev. Cell. 6, 673–684 (2004). 15130492 10.1016/s1534-5807(04)00107-8

[b24] Mal A., Sturniolo M., Schiltz R. L., Ghosh M. K. & Harter M. L. A role for histone deacetylase HDAC1 in modulating the transcriptional activity of MyoD: inhibition of the myogenic program. EMBO J. 20, 1739–1753 (2001). 11285237 10.1093/emboj/20.7.1739 PMC145490

[b25] Han J. . Apicidin, a histone deacetylase inhibitor, inhibits proliferation of tumor cells via induction of p21 WAF1 / Cip1 and gelsolin 1. Cancer Res. 60, 6068–6074 (2000). 11085529

[b26] Figeac N., Serralbo O., Marcelle C. & Zammit P. S. ErbB3 binding protein-1 (Ebp1) controls proliferation and myogenic differentiation of muscle stem cells. Dev. Biol. 386, 135–151 (2014). 24275324 10.1016/j.ydbio.2013.11.017

[b27] Halevy O. . Correlation of terminal cell cycle arrest of skeletal muscle with induction of p21 by MyoD. Science. 267, 1018–1021 (1995). 7863327 10.1126/science.7863327

[b28] Hagiwara H. . Histone deacetylase inhibitor trichostatin A enhances myogenesis by coordinating muscle regulatory factors and myogenic repressors. Biochem. Biophys. Res. Commun. 414, 826–831 (2011). 22019851 10.1016/j.bbrc.2011.10.036

[b29] Patel K. Follistatin. Int. J. Biochem. Cell Biol. 30, 1087–1093 (1998). 9785474 10.1016/s1357-2725(98)00064-8

[b30] Kalista S. . The type 1 insulin-like growth factor receptor (IGF-IR) pathway is mandatory for the follistatin-induced skeletal muscle hypertrophy. Endocrinology. 153, 241–253 (2012). 22087027 10.1210/en.2011-1687

[b31] Lokireddy S. . Myostatin promotes the wasting of human myoblast cultures through promoting ubiquitin-proteasome pathway-mediated loss of sarcomeric proteins. *A. J*. P. Cell Physiol. 301, C1316–C1324 (2011). 10.1152/ajpcell.00114.2011 21900687

[b32] Winbanks C. E. . Follistatin-mediated skeletal muscle hypertrophy is regulated by Smad3 and mTOR independently of myostatin. J. Cell Biol. 197, 997–1008 (2012). 22711699 10.1083/jcb.201109091 PMC3384410

[b33] Fan H. . Sulforaphane causes a major epigenetic repression of myostatin in porcine satellite cells. Epigenetics. 7, 1379–1390 (2012). 23092945 10.4161/epi.22609 PMC3528693

[b34] Ito A. . Induction of functional tissue-engineered skeletal muscle constructs by defined electrical stimulation. Sci. Rep. 4, 4781; 10.1038/srep04781 (2014). 24759171 PMC3998029

[b35] Yaffe D. & Saxel O. Serial passaging and differentiation of myogenic cells isolated from dystrophic mouse muscle. Nature. 270, 725–727 (1977). 563524 10.1038/270725a0

[b36] Ikeda K. . Effects of heat stimulation and L-ascorbic acid 2-phosphate supplementation on myogenic differentiation of artificial skeletal muscle tissue constructs. *J. Tissue Eng. Regen. Med*. in press. doi: 10.1002/term.2030. 10.1002/term.2030 26033935

[b37] Sato M., Ito A., Kawabe Y., Nagamori E. & Kamihira M. Enhanced contractile force generation by artificial skeletal muscle tissues using IGF-I gene-engineered myoblast cells. J. Biosci. Bioeng. 112, 273–278 (2011). 21646045 10.1016/j.jbiosc.2011.05.007

[b38] Hotta A. . Characterization of transient expression system for retroviral vector production. J. Biosci. Bioeng. 101, 361–368 (2006). 16716946 10.1263/jbb.101.361

