# Supplementary material for: In vitro drug testing based on contractile activity of C2C12 cells in an epigenetic drug model
Source: Sci Rep. 2017 Mar 16;7:44570. doi: 10.1038/srep44570 (PMC5353687; doi:10.1038/srep44570)
Supplement: Supplementary Information [file srep44570-s1.pdf]

Supplementary Information

In vitro drug testing based on contractile activity of C2C12 cells in an epigenetic drug model

Kazushi Ikeda[1], Akira Ito[2], Ryusuke Imada[2], Masanori Sato[2], Yoshinori Kawabe[2] & Masamichi Kamihira[1, 2]

[1]Graduate School of Systems Life Sciences, Kyushu University, 744 Motooka, Nishi-ku, Fukuoka 819-0395, Japan, [2]Department of Chemical Engineering, Faculty of Engineering, Kyushu University, 744 Motooka, Nishi-ku, Fukuoka 819-0395, Japan.

Correspondence and requests for materials should be addressed to M.K. (email: kamihira@chem-eng.kyushu-u.ac.jp)

Methods

Real-time PCR. Total RNA was isolated by RNAiso plus (Takara Bio, Shiga, Japan) on differentiation day 7, and then reverse transcribed with a reverse transcriptase (ReverTra Ace; Toyobo, Osaka, Japan) to generate cDNA samples. Real-time PCR was performed with a THUNDERBIRD SYBR qPCR RT Set (Toyobo) using a pair of primers [5'-CTACCCCCAATGTGTCCGTC-3' and 5'-GCTGTTGAAGTCGCAGGAGAC-3' for GAPDH; follistatin specific primer was designed by QuantiTect Primer Assays (Cat# QT00105483, Qiagen, Venlo, Netherlands)], and following profile: 40 cycles at 95°C for 5 s, 56°C for 20 s and 72°C 30 s. Thermocycling was performed in a final volume of 20 μL containing 1 μL of cDNA sample using LightCycler 1.5 (Roche, Basel, Switzerland). The relative gene expression was calculated in reference to the crossing point of GAPDH, and normalized by ΔΔCt method.

Immunofluorescent staining for α-actinin. Tissues were washed three times with phosphate-buffered saline (PBS) and fixed in 4% paraformaldehyde (PFA) for 15 min. They were then permeabilized in PBS containing 0.2% Triton X-100 for 15 min, washed three times with PBS, and blocked in PBS containing 1% (w/v) bovine serum albumin (BSA) for 30 min. The specimens were probed with a primary antibody against α-actinin (A-7811, monoclonal anti-α-actinin EA-53; Sigma-Aldrich, St. Louis, MO, USA) for 45 min. They were then washed three times with PBS and immersed in PBS containing 1% BSA, an Alexa546-conjugated secondary antibody (Life Technologies,

Carlsbad, CA, USA), and 4′,6-diamidino-2-phenylindole (DAPI) for 45 min. After washing three times with PBS, C2C12 cells or tissues were observed under a FV10i confocal laser-scanning microscope (Olympus, Tokyo, Japan), respectively.

Statistical analysis. Statistical comparisons were evaluated using the Mann-Whitney rank sum test, and the values of $P < 0.05$ were considered to be significantly different.

Figure legends

Supplementary Figure 1: Effects of epigenetic drugs on follistatin gene expression. Relative expression level of follistatin mRNA. C2C12 cells were treated with TSA (0.1 μM), VPA (1 mM), SB (5 mM), API (0.1 mM) or 5AC (0.01 mM) at day 4. Total RNA was isolated on day 7 and real time-PCR was performed with primers for follistatin and GAPDH. The data are expressed as mean ± SD of triplicate experiments. *$P < 0.05$ vs. control.

Supplementary Figure 2: Effects of follistatin gene transfer on sarcomere formation. Fluorescence microscopy images of α-actinin-positive myotubes (red) in the C2C12/FST tissue constructs at day 7. Nuclei were stained by DAPI (blue). Muscle tissue constructs were cultured in the presence (Dox+) or absence (Dox-) of doxycycline, and cultured with (EPS+) or without (EPS-) continuous EPS at amplitude of 0.3 V/mm, width of 4 ms, and frequency of 1Hz. Sarcomere structures were indicated by white arrows. Scale bars, 50 μm.

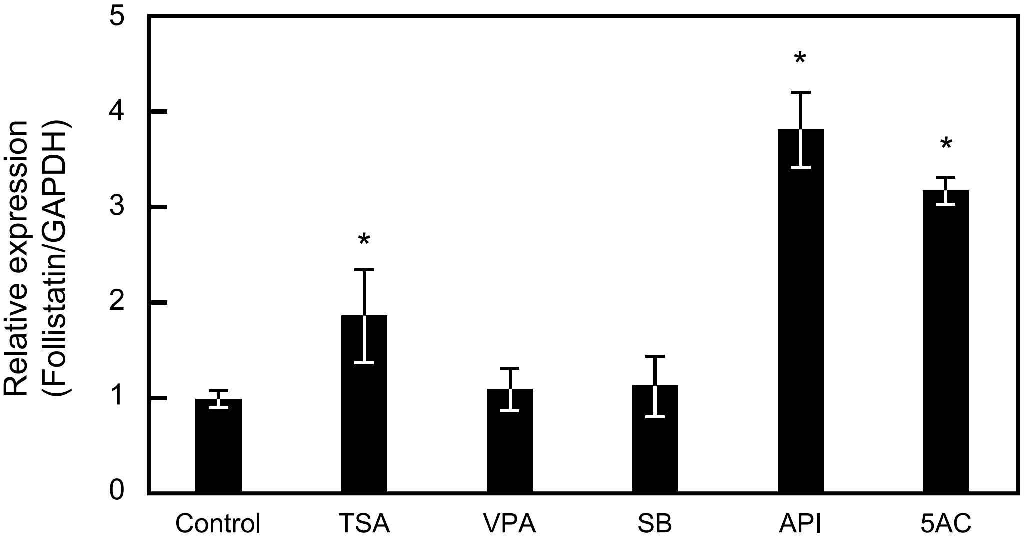

Supplementary Figure 1 Ikeda et al.

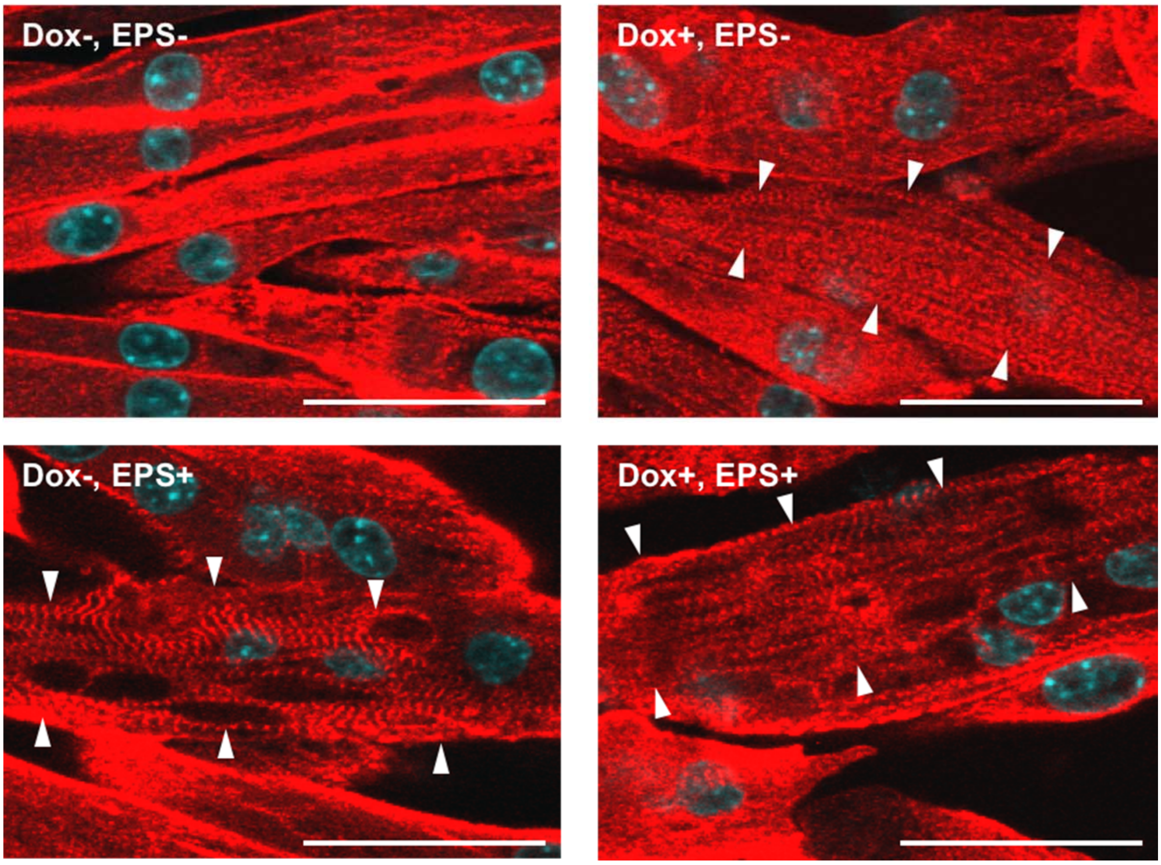

Supplementary Figure 2 Ikeda et al.
